# The identification of high-performing antibodies for Vacuolar protein sorting-associated protein 35 (hVPS35) for use in Western Blot, immunoprecipitation and immunofluorescence

**DOI:** 10.12688/f1000research.133696.1

**Published:** 2023-04-28

**Authors:** Riham Ayoubi, Maryam Fotouhi, Kathleen Southern, Peter S. McPherson, Carl Laflamme, NeuroSGC/YCharOS/EDDU collaborative group, ABIF consortium

**Affiliations:** 1 Department of Neurology and Neurosurgery, Structural Genomics Consortium, The Montreal Neurological Institute, McGill University, Montreal, Québec, H3A 2B4, Canada

**Keywords:** Uniprot ID Q96QK1, VPS35, hVPS35, Vacuolar protein sorting-associated protein 35, antibody characterization, antibody validation, Western Blot, immunoprecipitation, immunofluorescence

## Abstract

Vacuolar protein sorting-associated protein 35 is a subunit of the retromer complex, a vital constituent of the endosomal protein sorting pathway. The D620N mutation in the *VPS35* gene has been reported to be linked to type 17 Parkinson’s Disease progression, the exact molecular mechanism remains to be solved. The scientific community would benefit from the accessibility of validated and high-quality anti-hVPS35 antibodies. In this study, we characterized thirteen hVPS35 commercial antibodies for Western Blot, immunoprecipitation, and immunofluorescence using a standardized experimental protocol based on comparing read-outs in knockout cell lines and isogenic parental controls. We identified many high-performing antibodies and encourage readers to use this report as a guide to select the most appropriate antibody for their specific needs.

## Introduction

Vacuolar protein sorting-associated protein 35 (hVPS35) is a component of the retromer complex. ^1^ The retromer complex is a multimeric protein complex responsible for sorting transmembrane cargo from the endosome to the trans-Golgi network or plasma membrane, a pathway which is conserved across all eukaryotes. ^1^^,^^2^ Composed of two major subcomplexes, the cargo-selective complex and the membrane bound vacuolar protein sorting-associated protein (VPS) complex, hVPS35 serves as an essential component of the VPS complex where it mediates the recruitment of the retromer complex to the endosomal membrane. ^1^

Variants of the *VPS35* gene, otherwise referred to as *PARK17,* have recently been associated with the development of type 17 of Parkinson’s Disease (PD), among other neurodegenerative diseases. ^3^^–^^5^ A missense mutation in the gene, D620N, has been reported in numerous individuals and families with PD. ^6^^–^^12^ Further research is required to investigate the molecular mechanisms in which *VPS35* mutations induces neurodegeneration in PD. ^3^

Mechanistic studies would be greatly facilitated with the availability of high-quality antibodies. Here, we compared the performance of a range of commercially-available antibodies for hVPS35 and identified high-performing antibodies for Western Blot, immunoprecipitation and immunofluorescence, enabling biochemical and cellular assessment of hVPS35 properties and function.

## Results and discussion

Our standard protocol involves comparing readouts from wild-type (WT) and knockout (KO) cells. ^13^^–^^18^ The first step was to identify a cell line(s) that expresses sufficient levels of hVPS35 to generate a measurable signal. To this end, we examined the DepMap transcriptomics database to identify all cell lines that express the target at levels greater than 2.5 log _2_ (transcripts per million “TPM” + 1), which we have found to be a suitable cut-off (Cancer Dependency Map Portal, RRID:SCR_017655). Commercially available HAP1 cells expressed the hVPS35 transcript at RNA levels above the average range of cancer cells analyzed. Parental and *VPS35* KO HAP1 cells were obtained from Horizon Discovery ( Table 1).

**Table 1.** Summary of the cell lines used.

Institution	Catalog number	RRID (Cellosaurus)	Cell line	Genotype
Horizon Discovery	C631	CVCL_Y019	HAP1	WT
Horizon Discovery	HZGHC000863c012	CVCL_TX57	HAP1	*VPS35* KO

**Figure 1.**
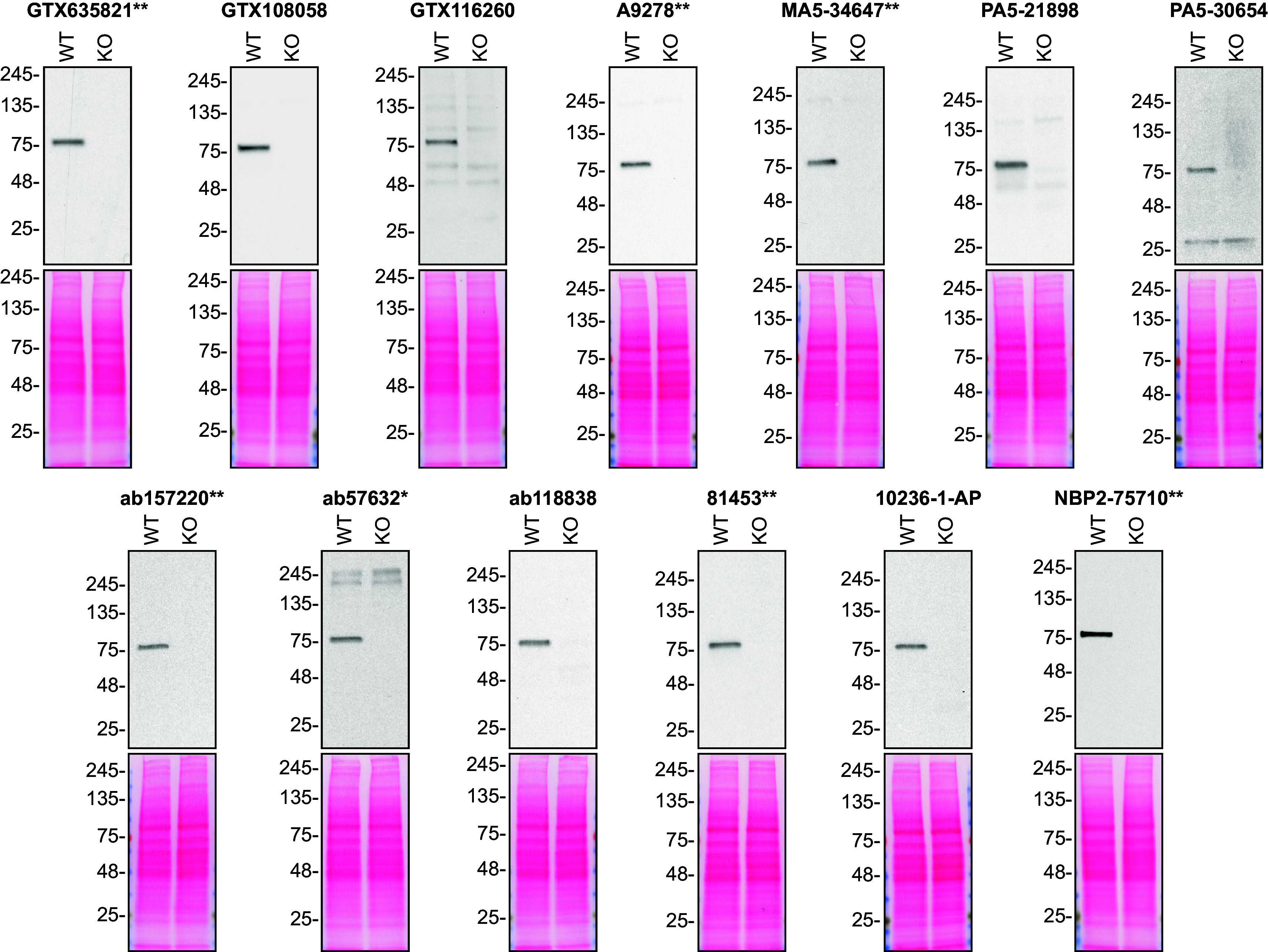
hVPS35 antibody screening by Western Blot. Lysates of HAP1 (WT and *VPS35* KO) were prepared and 20 μg of protein were processed for Western Blot with the indicated hVPS35 antibodies. The Ponceau stained transfers of each blot are presented to show equal loading of WT and KO lysates and protein transfer efficiency from the polyacrylamide gels to the nitrocellulose membrane. Antibody dilutions were chosen according to the recommendations of the antibody supplier. An exception was given for antibody 81453**, which was titrated to 1/500, as the signal was too weak when following the supplier’s recommendations. Antibody dilution used: GTX635821** at 1/1000, GTX108058 at 1/1000, GTX116260 at 1/1000, A9278** at 1/1000, MA5-34647** at 1/1000, PA5-21898 at 1/1000, PA5-30654 at 1/1000, ab157220** at 1/1000, ab57632* at 1/270, ab118838 at 1/900, 81453** at 1/500, 10236-1-AP at 1/500, NBP2-75710** at 1/1000. Predicted band size: 91 kDa. *=monoclonal antibody, **=recombinant antibody.

For Western Blot experiments, we resolved proteins from WT and *VPS35* KO cell extracts and probed them side-by-side with all antibodies in parallel ( Figure 1). ^14^^–^^18^

**Figure 2.**
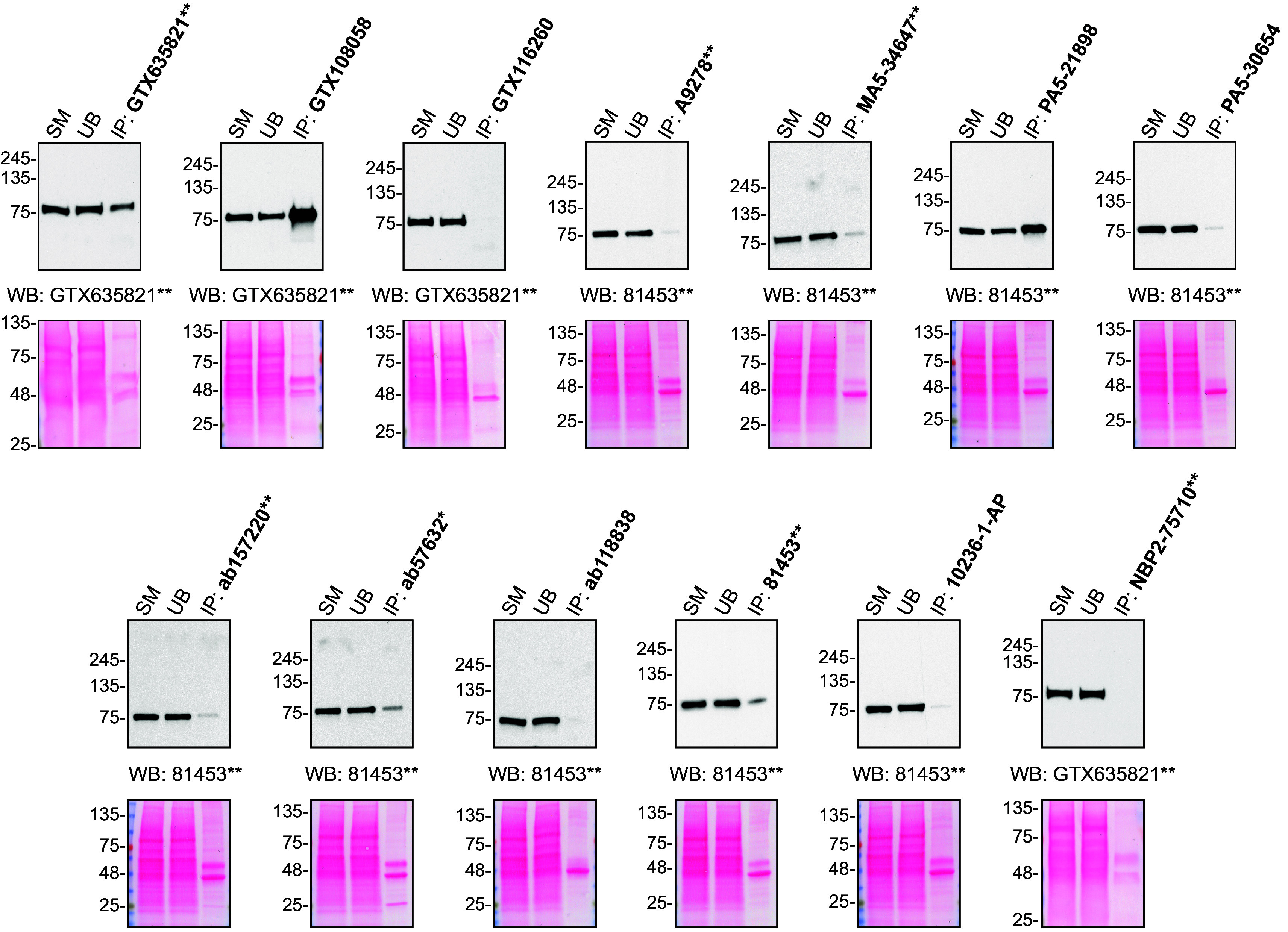
hVPS35 antibody screening by immunoprecipitation. HAP1 lysates were prepared, and IP was performed using 2.0 μg of the indicated hVPS35 antibodies pre-coupled to Dynabeads protein A or protein G. Samples were washed and processed for Western Blot with the indicated hVPS35 antibody. For Western Blot, GTX635821** and 81453** were used at 1/1000 and 1/500, respectively. The Ponceau stained transfers of each blot are shown. SM=2% starting material; UB=2% unbound fraction; IP=immunoprecipitate. *=monoclonal antibody, **=recombinant antibody.

For immunoprecipitation experiments, we used the antibodies to immunopurify hVPS35 from HAP1 cell extracts. The performance of each antibody was evaluated by detecting the hVPS35 protein in extracts, in the immunodepleted extracts and in the immunoprecipitates ( Figure 2). ^14^^–^^18^

**Figure 3.**
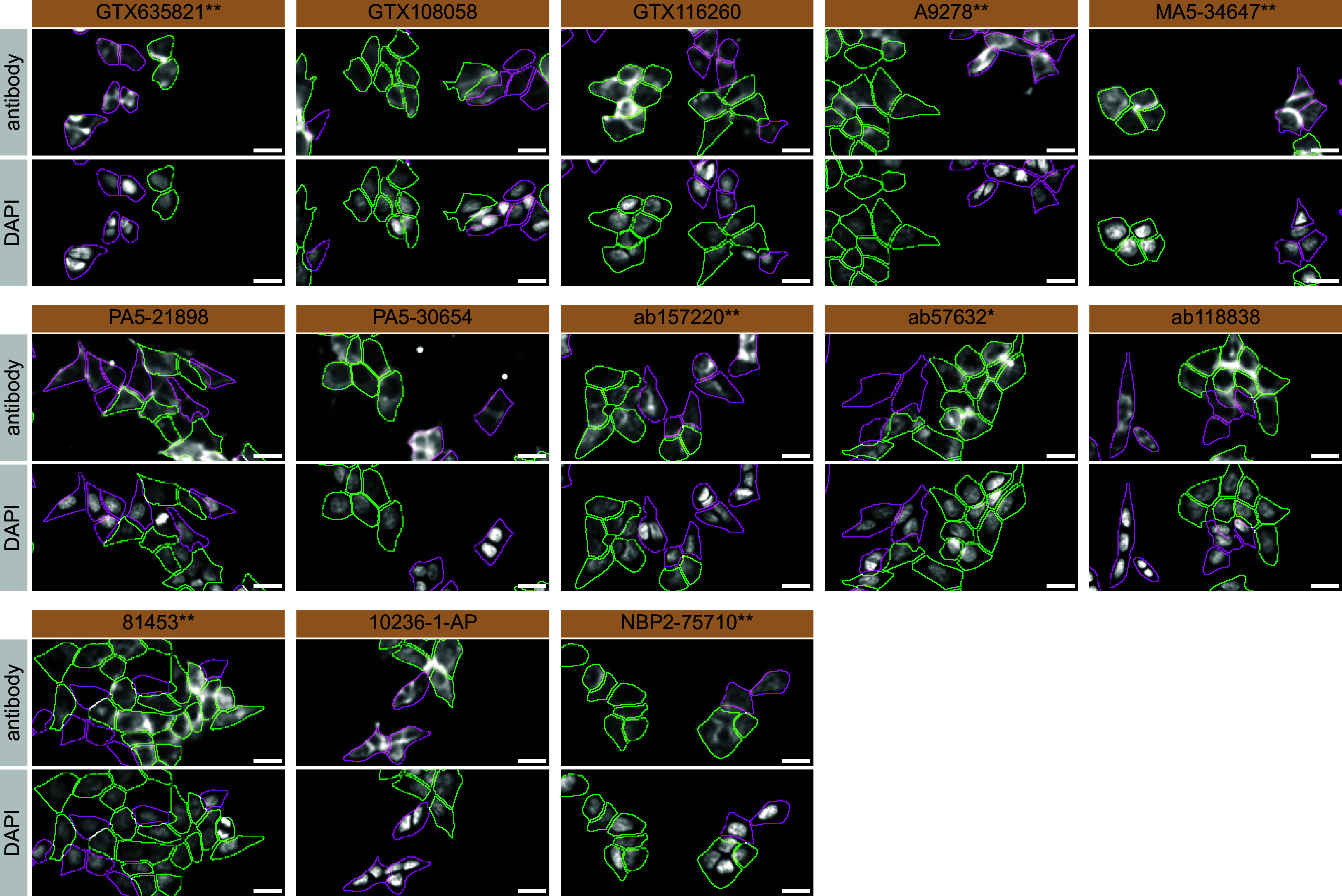
hVPS35 antibody screening by immunofluorescence. HAP1 WT and *VPS35* KO cells were labelled with a green or a far-red fluorescent dye, respectively. WT and KO cells were mixed and plated to a 1:1 ratio in a 96-well plate with a glass bottom. Cells were stained with the indicated hVPS35 antibodies and with the corresponding Alexa-fluor 555 coupled secondary antibody including DAPI. Acquisition of the blue (nucleus-DAPI), green (WT), red (antibody staining) and far-red (KO) channels was performed. Representative images of the merged blue and red (grayscale) channels are shown. WT and KO cells are outlined with green and magenta dashed line, respectively. Antibody dilutions were chosen according to the recommendations of the antibody supplier. When the concentration was not indicated by the supplier, which was the case for antibodies GTX116260, A9278**, MA5-34647**, PA5-30654, 81453** and NBP2-75710**, we tested the antibodies at 1/600, 1/400, 1/1000, 1/60, 1/60 and 1/500, respectively. At these concentrations, the signal from each antibody was in the range of detection of the microscope used. Antibody dilution used: GTX635821** at 1/1000, GTX108058 at 1/600, GTX116260 at 1/600, A9278** at 1/400, MA5-34647** at 1/1000, PA5-21898 at 1/100, PA5-30654 at 1/60, ab157220** at 1/500, ab57632* at 1/1000, ab118838 at 1/1000, 81453** at 1/60, 10236-1-AP at 1/50, NBP2-75710** at 1/500. Bars = 10 μm. *=monoclonal antibody, **=recombinant antibody.

For immunofluorescence, as described previously, antibodies were screened using a mosaic strategy. ^19^ In brief, we plated WT and KO cells together in the same well and imaged both cell types in the same field of view to reduce staining, imaging and image analysis bias ( Figure 3).

In conclusion, we have screened hVPS35 commercial antibodies by Western Blot, immunoprecipitation and immunofluorescence and identified several high-quality antibodies under our standardized experimental conditions. The underlying data can be found of Zenodo. ^20^^,^^21^

## Methods

### Antibodies

All hVPS35 antibodies are listed in Table 2, together with their corresponding Research Resource Identifiers, or RRID, to ensure the antibodies are cited properly. ^22^ Peroxidase-conjugated goat anti-rabbit and anti-mouse antibodies are from Thermo Fisher Scientific (cat. number 62-6120 and 65-6520). Alexa-555-conjugated goat anti-rabbit and anti-mouse secondary antibodies are from Thermo Fisher Scientific (cat. number A21429 and A21424).

**Table 2.** Summary of the hVPS35 antibodies tested.

Company	Catalog number	Lot number	RRID (Antibody Registry)	Clonality	Clone ID	Host	Concentration (μg/μL)	Vendors recommended applications
GeneTex	GTX635821 **	44151	AB_2888583	recombinant-mono	HL1017	rabbit	1.00	Wb, IF
GeneTex	GTX108058	43950	AB_1241448	polyclonal	-	rabbit	0.66	Wb, IF
GeneTex	GTX116260	40282	AB_10626418	polyclonal	-	rabbit	0.64	Wb, IP
Abclonal	A9278 **	4000001509	AB_2863704	recombinant-mono	ARC1509	rabbit	0.37	Wb
Thermo Fisher Scientific	MA5-34647 **	XD3571015	AB_2848555	recombinant-mono	JB33-82	rabbit	1.00	Wb
Thermo Fisher Scientific	PA5-21898	XE3572719	AB_11153540	polyclonal	-	rabbit	0.66	Wb, IF
Thermo Fisher Scientific	PA5-30654	XD3572719D	AB_2548128	polyclonal	-	rabbit	0.64	Wb, IP
Abcam	ab157220 **	GR117932	AB_2636885	recombinant-mono	EPR11501(B)	rabbit	0.11	Wb, IF
Abcam	ab57632 *	GR3330429	AB_946126	monoclonal	2D3	mouse	0.27	Wb
Abcam	ab118838	GR250228	AB_2923524 ^1^	polyclonal	-	rabbit	0.90	Wb, IF
Cell Signaling Technology	81453 **	1	AB_2923525 ^1^	recombinant-mono	E6S4l	rabbit	0.06	Wb, IP
Proteintech	10236-1-AP	22564	AB_2215216	polyclonal	-	rabbit	0.26	Wb, IP, IF
Bio-Techne	NBP2-75710 **	HP0606	AB_2923523 ^1^	recombinant-mono	JB33-82	rabbit	1.00	Wb

Wb=Western blot; IF= immunofluorescence; IP=immunoprecipitation.

* = monoclonal antibody.

** = recombinant antibody.

^1^ refers to RRID recently added to the Antibody Registry (on February 2023), they will be available on their website in the coming weeks.

### Cell culture

Both HAP1 WT and *VPS35* KO cell lines used are listed in Table 1, together with their corresponding RRID, to ensure the cell lines are cited properly. ^23^ Cells were cultured in DMEM high-glucose (GE Healthcare cat. number SH30081.01) containing 10% fetal bovine serum (Wisent, cat. number 080450), 2 mM L-glutamate (Wisent cat. number 609065), 100 IU penicillin and 100 μg/mL streptomycin (Wisent cat. number 450201).

### Antibody screening by Western Blot

Western Blots were performed as described in our standard operating procedure. ^24^ HAP1 WT and *VPS35* KO were collected in RIPA buffer (25mM Tris-HCl pH 7.6, 150mM NaCl, 1% NP-40, 1% sodium deoxycholate, 0.1% SDS) (Thermo Fisher Scientific, cat. number 89901) supplemented with 1x protease inhibitor cocktail mix (MilliporeSigma, cat. number 78429). Lysates were sonicated briefly and incubated for 30 min on ice. Lysates were spun at ~110,000 x g for 15 min at 4°C and equal protein aliquots of the supernatants were analyzed by SDS-PAGE and Western Blot. BLUelf prestained protein ladder from GeneDireX (cat. number PM008-0500) was used.

Western Blots were performed with pre-cast mini 4-15% gradient polyacrylamide gels from Bio-Rad (cat. number 4561084) and transferred onto nitrocellulose membranes. Proteins on the blots were visualized with Ponceau S staining (Thermo Fisher Scientific, cat. number BP103-10) which is scanned to show together with individual Western Blot. Blots were blocked with 5% milk for 1 hr, and antibodies were incubated overnight at 4°C with 5% bovine serum albumin (BSA) (Wisent, cat. number 800-095) in TBS with 0,1% Tween 20 (TBST) (Cell Signaling Technology, cat. number 9997). Following three washes with TBST, the peroxidase conjugated secondary antibody was incubated at a dilution of ~0.2 μg/mL in TBST with 5% milk for 1 hr at room temperature followed by three washes with TBST. Membranes were incubated with Pierce ECL from Thermo Fisher Scientific (cat. number 32106) prior to detection with the HyBlot CL autoradiography films from Denville (cat. number 1159T41).

### Antibody screening by immunoprecipitation

Immunoprecipitation was performed as described in our standard operating procedure. ^25^ Antibody-bead conjugates were prepared by adding 2 μg to 500 μL of Pierce IP Lysis Buffer from Thermo Fisher Scientific (cat. number 87788) in a 1.5 mL microcentrifuge tube, together with 30μL of Dynabeads protein A- (for rabbit antibodies) or protein G- (for mouse antibodies) from Thermo Fisher Scientific (cat. number 10002D and 10004D, respectively). Tubes were rocked for ~1 hr at 4°C followed by two washes to remove unbound antibodies.

HAP1 WT were collected in Pierce IP buffer (25 mM Tris-HCl pH 7.4, 150 mM NaCl, 1 mM EDTA, 1% NP-40 and 5% glycerol) (Thermo Fisher Scientific, cat. number 87788) supplemented with protease inhibitor (MilliporeSigma, cat. number P8340). Lysates were rocked for 30 min at 4°C and spun at 110,000 x g for 15 min at 4°C. 0.5 mL aliquots at 2.0 mg/mL of lysate were incubated with an antibody-bead conjugate for ~2 hrs at 4°C. The unbound fractions were collected, and beads were subsequently washed three times with 1.0 mL of IP lysis buffer and processed for SDS-PAGE and Western Blot on a pre-cast mini 4-15% gradient polyacrylamide gels. Prot-A: HRP (MilliporeSigma, cat. number P8651) was used as a secondary detection system at a concentration of 0.3 μg/mL.

### Antibody screening by immunofluorescence

Immunofluorescence was performed as described in our standard operating procedure. ^14^^–^^19^ HAP1 WT and *VPS35* KO were labelled with a green and a far-red fluorescence dye, respectively. The fluorescent dyes used are from Thermo Fisher Scientific (cat. number C2925 and C34565). The nuclei were labelled with DAPI (Thermo Fisher Scientific, cat. Number D3571) fluorescent stain. WT and KO cells were plated in 96 well glass plates (Perkin Elmer, cat. number 6055300) as a mosaic and incubated for 24 hrs in a cell culture incubator at 37 ^o^C, 5% CO _2_. Cells were fixed in paraformaldehyde (PFA) (Beantown chemical, cat. number 140770-10ml) in phosphate buffered saline (PBS) (Wisent, cat. number 311-010-CL) for 15 min at room temperature and then washed 3 times with PBS. Cells were permeabilized in PBS with 0,1% Triton X-100 (Thermo Fisher Scientific, cat. number BP151-500) for 10 min at room temperature and blocked with PBS with 5% BSA, 5% goat serum (Gibco, cat. number 16210-064) and 0.01% Triton X-100 for 30 min at room temperature. Cells were incubated with IF buffer (PBS, 5% BSA, 0,01% Triton X-100) containing the primary hVPS35 antibodies overnight at 4°C. Cells were then washed 3 × 10 min with IF buffer and incubated with corresponding Alexa Fluor 555-conjugated secondary antibodies in IF buffer at a dilution of 1.0 μg/mL for 1 hr at room temperature with DAPI. Cells were washed 3 × 10 min with IF buffer and once with PBS.

Images were acquired on an ImageXpress micro widefield high-content microscopy system (Molecular Devices), using a 20x/0.95 NA water objective lens and scientific CMOS camera (16-bit, 1.97mm field of view), equipped with 395, 475, 555 and 635 nm solid state LED lights (Lumencor Aura III light engine) and bandpass emission filters (432/36 nm, 520/35 nm, 600/37 nm and 692/40 nm) to excite and capture fluorescence emission for DAPI, CellTracker ^TM^ Green, Alexa fluor 555 and CellTracker ^TM^ Red, respectively. Images had pixel sizes of 0.68 x 0.68 microns. Exposure time was set with maximal (relevant) pixel intensity ~80% of dynamic range and verified on multiple wells before acquisition. Since the IF staining varied depending on the primary antibody used, the exposure time was set using the most intensely stained well as reference. Frequently, the focal plane varied slightly within a single field of view. To remedy this issue, a stack of three images per channel was acquired at a z-interval of 4 microns per field and best focus projections were generated during the acquisition (MetaExpress v6.7.1, Molecular Devices). Segmentation was carried out on the projections of CellTracker ^TM^ channels using CellPose v1.0 on green (WT) and far-red (KO) channels, using as parameters the ‘cyto’ model to detect whole cells, and using an estimated diameter tested for each cell type, between 15 and 20 microns. ^26^ Masks were used to generate cell outlines for intensity quantification. Figures were assembled with Adobe Photoshop (version 24.1.2) to adjust contrast then assembled with Adobe Illustrator (version 27.3.1).

## Data Availability

Zenodo: Antibody Characterization Report for hVPS35, https://doi.org/10.5281/zenodo.7671730. ^20^ Zenodo: Dataset for the hVPS35 antibody screening study, https://doi.org/10.5281/zenodo.7795779. ^21^
