# Supplementary material for: Identification of high-performing antibodies for Vacuolar protein sorting-associated protein 35 (hVPS35) for use in Western Blot, immunoprecipitation and immunofluorescence
Source: F1000Res. 2023 Oct 16;12:452. Originally published 2023 Apr 28. [Version 2] doi: 10.12688/f1000research.133696.2 (PMC10905012; doi:10.12688/f1000research.133696.2)
Supplement: Peer review history

# Reviewer report: Reviewer response for version 2

**DOI:** 10.5256/f1000research.157173.r234849

**Published:** 2024-02-09

**Authors:** Emily Sontag

**Affiliations:** 1 Marquette University, Milwaukee, Wisconsin, USA

The article characterizes 13 different commercial antibodies to the human VPS35 protein, which is associated with Parkinson's disease. This information is highly valuable to the field, but a few clarifications will assist readers in interpreting the data and using it to make decisions on which reagents to use in their own experiments. 

 The amendments provide additional information about PARK17, but introduce a bit of confusion. In the third sentence of the first paragraph of the introduction, it is unclear if the retromer complex or the hVPS35 is composed of 2 subcomplexes. Additionally, the first sentence of the second paragraph of the introduction is confusing as it appears that the sentence is repeated within itself. The definition of a high-performance for each application is very clearly stated. 

 While the authors are clear on why they do not offer analyses or recommendations, it would be very helpful to include quantification of the Western blots from the IP experiments and the intensity values of the immunofluorescence. Several of the antibodies do not appear to meet the threshold for high-performance by those techniques (10% of input and a 1.5-fold increase in signal, respectively). The measured values would aid experts in interpreting the data and determining which antibodies meet the standard of a high-performing antibody.

 Additionally, it is unclear why the GTX635821 and 81453 antibodies were chosen for the Western blot analysis of the IP experiments. The NBP2-75710 antibody appears to give the best signal on the Western blot screening in Figure 1. The legend for Figure 3 states that the representative images of the blue and red channels are merged, but they are shown as separate grayscale images. 

 The article is scientifically sound and contributes to the field, but could be improved with minor revisions.

Are sufficient details of methods and materials provided to allow replication by others?

Yes

Is the rationale for creating the dataset(s) clearly described?

Yes

Are the datasets clearly presented in a useable and accessible format?

Partly

Are the protocols appropriate and is the work technically sound?

Yes

Reviewer Expertise:

Biochemistry, Cell Biology, protein homeostasis, ESCRT and VPS protein function.

I confirm that I have read this submission and believe that I have an appropriate level of expertise to confirm that it is of an acceptable scientific standard.

# Reviewer report: Reviewer response for version 2

**DOI:** 10.5256/f1000research.157173.r215700

**Published:** 2023-12-21

**Authors:** Maria-Paz Marzolo

**Affiliations:** 1 Pontificia Universidad Catolica de Chile, Santiago, Santiago Metropolitan Region, Chile; 2 Laboratorio de Tráfico Intracelular y Señalización, Departamento de Biología Celular y Molecular, Facultad de Ciencias Biológicas, Pontificia Universidad Catolica de Chile, Santiago, Santiago Metropolitan Region, Chile

In this new revised version, the authors have included the references suggested, and they have improved the introduction with a detailed explanation of retromer complex, its implication in Parkinson´s disease, and the definition of a high-performing antibody. The section is now clear and complete.

 This is a well-performed study, with relevant data to be considered for researchers working on retromer functions and expression, which, as was suggested by our initial review, would benefit by including more than one cell line. However, the authors explain the study's limitations in terms of using only one cell line. We think the study has value in itself and contributes to the field.

Are sufficient details of methods and materials provided to allow replication by others?

Partly

Is the rationale for creating the dataset(s) clearly described?

Partly

Are the datasets clearly presented in a useable and accessible format?

Partly

Are the protocols appropriate and is the work technically sound?

Partly

Reviewer Expertise:

Cell Biology, neurobiology, membrane trafficking, cell polarity, signaling pathways.

I confirm that I have read this submission and believe that I have an appropriate level of expertise to confirm that it is of an acceptable scientific standard.

# Reviewer report: Reviewer response for version 1

**DOI:** 10.5256/f1000research.146706.r191098

**Published:** 2023-09-07

**Authors:** Maria-Paz Marzolo, Vania Macias

**Affiliations:** 1 Pontificia Universidad Catolica de Chile, Santiago, Santiago Metropolitan Region, Chile; 2 Laboratorio de Tráfico Intracelular y Señalización, Departamento de Biología Celular y Molecular, Facultad de Ciencias Biológicas, Pontificia Universidad Catolica de Chile, Santiago, Santiago Metropolitan Region, Chile; 3 Cell and Molecular Biology, Pontifical Catholic University of Chile, Santiago, Santiago Metropolitan Region, Chile

The article is a data note that evaluates different commercial antibodies for the human Vacuolar protein sorting-associated protein 35 (hVPS35),a. subunit of the Retromer complex, also composed of VPS26 and VPS29 subunits. Retromer is involved in several cargo sorting processes at the endosomes, regulating endosome to plasma membrane trafficking of cargoes and the endosome to Golgi apparatus retrograde transport of cargoes such as the mannose-6-phosphate receptors. Mutations in Retromer are associated with neurodegenerative conditions, such as Parkinson's Disease (PD). This article is particularly relevant as a data note for evaluating different commercial antibodies for VPS35 detection, but some mistakes should be addressed.

 In the Abstract and the Introduction, it is mentioned a type 17 Parkinson's disease progression. The *VPS35* gene is the *PARK17*, but there are not 17 types of PD. The authors could check the PARK genes that historically are the genes that have been linked to PD. Reference 1 can be complemented with other articles; I suggest including the articles:

 Haft, C. R., de la Luz Sierra, M., Bafford, R., Lesniak, M. A., Barr, V. A., & Taylor, S. I. (2000). Human orthologs of yeast vacuolar protein sorting proteins Vps 26, 29, and 35: Assembly into multimeric complexes. Molecular Biology of the Cell, 11, 4105–4116.

 Seaman, M. N. J., Marcusson, E. G., Cereghino, J. L., & Emr, S. D. (1997). Endosome to Golgi retrieval of the vacuolar protein sorting receptor,

 Vps10p requires the function of the VPS29, VPS30, and VPS35 gene products. Journal of Cell Biology, 137, 79–92.

 Seaman, M. N. J., McCaffery, J. M., & Emr, S. D. (1998). A membrane coat complex essential for endosome-to-Golgi retrograde transport in yeast. Journal of Cell Biology, 142, 665–681.

 Seaman, M. N. J. (2004). Cargo-selective endosomal sorting for retrieval to the Golgi requires a retromer. Journal of Cell Biology, 165, 111–122. https://doi.org/10.1083/jcb.20031 2034

**Is the rationale for creating the dataset(s) clearly described?**

 The study is relevant to the field. It is necessary to characterize and find reliable antibodies that can detect the VPS35, without false positives, for describing and ascribing different and eventually new retromer functions and cargoes. The authors established that the main AIM of the data note is to use it as a guide to select the appropriate antibody for the research. The guide could help scientists in the field to invest in quality antibodies, saving time, money, and other resources in the characterization of antibodies that, in the end, will be useless. This AIM is accomplished; as a guide, this article gives useful information. However, it could be improved by discussing the reliability of the antibodies tested and the pertinence of the results obtained.

**Are the protocols appropriate, and is the work technically sound?**

 The authors analyze the antibody's performance in three techniques widely utilized in cell biology: western blot, immunoprecipitation, and immunofluorescence. These are techniques often used in the field, including membrane trafficking studies and neurobiology studies. Nevertheless, the results could be improved.

 The authors decided to test the antibodies in the carcinogenic cell line HAP1 should explore other non-carcinogenic cell lines as well as neuronal cell lines considering that many mutants of VPS35 are associated with neurodegenerative diseases, including PD. Moreover, even among cells of the same species, antibody performance also depends on the cellular type; therefore, it would be desirable to test the antibodies in a neuron-like cell line as H4, SH-Sy5Y, to mention some.

 In the western blot experiments, there are unspecific bands in the WT and KO lines for GTX116260, PA5-30654, and ab57632*. This should be remarked in an additional table or Table 2 for scientists to evaluate the pertinence of these antibodies.

 In the immunofluorescence, the signal shown does not correlate with an expected signal of Retromer following the VPS35 subunit. It should show a vesicular pattern with identifiable structures. This could be correlated with other endosome markers or cargos such as mannose-6-phosphate, the GTPase Rab7, or the expression of a GFP-tag VPS35. Besides, authors should consider testing other fixations as methanol and performing negative controls in non-permeabilized cells. I wonder if 20X is the ideal magnification for the analysis because the label is very diffused. However, comparing the WT and KO lines in the same field is a great idea.

**Are sufficient details of methods and materials provided to allow replication by others?**

 The methods are clear and detailed. However, the authors could explain why, for the western blots, they decided to block with milk and then incubate the primary antibody in BSA. For example. for some antibodies, the BSA is also required for blocking. A clarification on the concept of high-performing antibodies and what it entails should be included.

**Are the datasets presented in a usable and accessible format?**

 The datasets are clearly and accessibly presented, but Table 2 could include the performance of the antibodies in the different techniques and if the antibodies have been cited in other articles.

Are sufficient details of methods and materials provided to allow replication by others?

Partly

Is the rationale for creating the dataset(s) clearly described?

Partly

Are the datasets clearly presented in a useable and accessible format?

Partly

Are the protocols appropriate and is the work technically sound?

Partly

Reviewer Expertise:

Cell Biology, neurobiology, membrane trafficking, cell polarity, signaling pathways.

We confirm that we have read this submission and believe that we have an appropriate level of expertise to confirm that it is of an acceptable scientific standard, however we have significant reservations, as outlined above.

## References

[1] : Human orthologs of yeast vacuolar protein sorting proteins Vps26, 29, and 35: assembly into multimeric complexes. *Mol Biol Cell* .2000;11(12) : 10.1091/mbc.11.12.4105 4105-16 10.1091/mbc.11.12.4105 11102511 PMC15060

[2] : Endosome to Golgi retrieval of the vacuolar protein sorting receptor,Vps10p requires the function of the VPS29, VPS30, and VPS35 gene products. *Journal of Cell Biology* .1997;79-92 9105038 10.1083/jcb.137.1.79PMC2139870

[3] : A membrane coat complex essential for endosome-to-Golgi retrograde transport in yeast. *J Cell Biol* .1998;142(3) : 10.1083/jcb.142.3.665 665-81 10.1083/jcb.142.3.665 9700157 PMC2148169

[4] : Cargo-selective endosomal sorting for retrieval to the Golgi requires retromer. *J Cell Biol* .2004;165(1) : 10.1083/jcb.200312034 111-22 10.1083/jcb.200312034 15078902 PMC2172078

# Response

**Published:** 2023-10-06

**Authors:** Kathleen Southern

**Affiliations:** McGill University, Canada

Thank you for your review and feedback. We will be addressing your comments and submitting a new version of the article, with many of your suggestions included.

 To the abstract and introduction, we have removed the mention of type 17 Parkinson’s disease. Furthermore, in the introduction we have included information to mention other *PARK* genes that have been identified in research, including *PARK17*. We are always looking to improve on our articles and hope this satisfies your feedback!

 In regard to your comment on the rationale for creating the dataset, we understand that you believe it would enhance the quality of the article if we discussed the performance of the antibodies tested and reported their specificity or selectivity in regards to the results. In response to this feedback, it’s important to clarify that YCharOs does not engage in result analysis nor do we offer explicit antibody recommendations. We’re not experts of these proteins and prefer to remain impartial which is why we carry out the study without interpreting the results. The purpose of YCharOS is to deliver antibody characterization reports as a public good collection to benefit biomedical research as a whole. It is for this reason that we chose to publish our articles using the Data Note format, as it does not require result analysis but rather presents KO characterization data for experts of the individual targets to further their studies and projects. We have found that, for the most part, scientists viewing our articles have the expertise to the characterization data independently, enabling them to make informed choices regarding the most suitable antibodies for their specific experimental needs. We understand that this point has not been made clear within the article, which is why we have added a new paragraph to the Results and Discussion section, explaining our reasonings as to why we do not recommend or score the antibodies tested.

 In terms of cell lines, at this point, testing and validating the antibodies in various neuronal cell lines is outside the scope of our standard protocol and study. We utilize the Cancer Dependency Map (DepMap) as a guide to select a cell line with adequate levels of protein of interest. That being said, we hope to revisit all Parkinson’s disease- related protein targets in the future by quantifying the antibody performance on a larger scale and analyzing protein expression in various neuronal cell lines.

 In terms of evaluating the antibodies you mentioned in Western blot, as previously mentioned, we believe scientists viewing the article will have the expertise to see that the antibodies mentioned are non-selective using our current experimental set-up.

 The reason as to why different fixation methods were not used, is because with our current workflow we identified antibodies that specifically target hVPS35 by immunofluorescence (IF). It is only in the case that no successful antibodies were identified that we consider other fixation protocols such as methanol.

 Furthermore, our goal for the IF application is not to identify the subcellular distribution of hVPS35, but rather demonstrate to the scientific community which of the antibodies perform successfully in IF. Meaning, we look for antibodies that immunolocalizes the target protein by generating a fluorescence signal in WT cells that is at least 1.5-fold higher than the signal in KO cells (1). Using a 20X magnification allows us to evaluate the antibodies selectivity but does not have the resolution to dive into the subcellular distribution of the antibodies. With our reports, the experts now know which antibodies are successful in identifying VPS35, and can further their research by identifying subcellular distribution utilizing organelle markers.

 We intend on clarifying this factor in our FAQ section on our YCharOS gateway.

 After performing our standard operating procedure for around 80 protein targets and 800 antibodies, we haven’t seen a difference in results when blocking with milk or BSA. Furthermore, Milk was chosen as a blocking solution as it doesn’t get contaminated as easily, allowing us to repeat the experiment, if necessary. 

 We agree that the definition of a high-performing antibody is not made evident in the first version of the article. In the introduction of the new article version to be submitted shortly, we have included a small description of what high-performing antibody signifies in each application. You can also visit an article written by Riham et al. which provides an in-depth description on what a successful antibody looks like in each application (Box 1) (1)

 We don’t look at antibody performance in individual reports, but we do in a separate study, looking at the overall performance of antibodies, compared to their use in literature (1).

 1. Ayoubi R, Ryan J, Biddle MS, Alshafie W, Fotouhi M, Bolivar SG, et al. Scaling of an antibody validation procedure enables quantification of antibody performance in major research applications. eLife. 2023;12:RP91645.
